# Supplementary material for: Knowledge on Stroke Recognition and Management among Emergency Department Healthcare Professionals in the Republic of Cyprus
Source: Healthcare (Basel). 2023 Dec 29;12(1):77. doi: 10.3390/healthcare12010077 (PMC10778653; doi:10.3390/healthcare12010077)
Supplement: Supplementary file 1 [file healthcare-12-00077-s001.zip › healthcare-2740178-supplementary.pdf]

Supplementary File S1 (SF1): Questionnaire for assessing knowledge on stroke recognition and management among Emergency Department Healthcare Professionals in the Republic of Cyprus

Part A

**Sociodemographic and employment characteristics**						
1. Age	20-29	30-39		40-49	50-60	>60
2. Gender	Male			Female		
3. Profession	Nurse			Physician		
4. Educational attainment	Diploma of Nursing School	Bachelor's Degree		Nursing specialty program		Master's Degree
5. Years of work experience	<1	1-5	6-10	11-20	21-30	>30
6. Name/ region of the hospital……………………… <br>Type of hospital	Public			Private		
7. Prior education or training relevant to stroke care	Yes			No		
8. If you had any prior education or training relevant to stroke care, what was the source of such education or training? (please circle all that apply)	Class (undergraduate level)	Class (postgraduate level)	Brief presentation on stroke care	Congress/ educational work-shop	Leaflet/ other printed material	Self-guided study

Part B

Knowledge questions - Please circle the correct answer:		
1. Every patient presenting with stroke should be treated as a medical emergency, whether eligible for thrombolysis or not		
A. RIGHT	B. WRONG	C. DON'T KNOW
2. Stroke represents a life-threatening situation		
A. RIGHT	B. WRONG	C. DON'T KNOW
3. EDs play a vital role in the early recognition of stroke and the timely commencement of treatment		
A. RIGHT	B. WRONG	C. DON'T KNOW
4. EMS personnel should inform ED about the transfer of a patient with a probable stroke		
A. RIGHT	B. WRONG	C. DON'T KNOW
5. NIHSS is the proposed scale for stroke severity assessment		
A. RIGHT	B. WRONG	C. DON'T KNOW
6. A patient presenting to the ED with stroke is most likely to experience which of the following symptoms?<br>i) Tremor, dizziness, vomiting<br>ii) Altered level of consciousness, tachypnea, cyanosis<br>iii) Unilateral arm or leg weakness or face drooping or difficulty speaking		
7. Which of the following can mimic stroke?		

i) Hypoglycemia<br>ii) Hyperkalemia<br>iii) Heat stroke<br>iv) Pulmonary embolism
8. Brain MRI is the recommended diagnostic modality to differentiate between ischemic and hemorrhagic stroke<br>A. RIGHT B. WRONG C. DON'T KNOW
9. In a patient with suspected stroke, clinical examination and history taking can securely differentiate between ischemic and hemorrhagic stroke<br>A. RIGHT B. WRONG C. DON'T KNOW
10. 60 minutes is the maximum time allowed from the arrival of the patient at the ED and the commencement of diagnostic examinations<br>A. RIGHT B. WRONG C. DON'T KNOW
11. A patient with suspected stroke, regardless of severity and neurological deficit, must be placed in bed, supine<br>A. RIGHT B. WRONG C. DON'T KNOW
12. In patients with suspected stroke, blood pressure should be measured on both arms and a finger stick glucose test performed<br>A. RIGHT B. WRONG C. DON'T KNOW
13. Hypotension and hypovolemia should be treated before starting thrombolysis<br>A. RIGHT B. WRONG C. DON'T KNOW
14. Thrombolysis must be administered to eligible stroke patients within a time-window of 3 hours<br>A. RIGHT B. WRONG C. DON'T KNOW
15. Patients aged over 80 years should be excluded from thrombolysis

A. RIGHT B. WRONG C. DON'T KNOW

16. In a stroke patient about to receive thrombolysis, the maximum acceptable body temperature is:

A. 37◦C B. 37,5 ◦C C. 38 ◦C D. 38,5 ◦C

17. The lowest acceptable blood glucose level for a patient about to receive thrombolysis is 60 mg/dl

A. RIGHT B. WRONG C. DON'T KNOW

18. If the patient that is about to receive thrombolysis demonstrates an oxygen saturation of <94%, we administer oxygen via nasal cannula and proceed with thrombolysis as planned

A. RIGHT B. WRONG C. DON'T KNOW

19. What is the recommended dose of rt-PA in a stroke patient?

A. 0,9mg/kg B. 90 mg/kg C. 0,6 mg/kg D. 1,3 mg/kg

20. What is the maximum acceptable blood pressure before administering thrombolysis?

A. 200/115 B. 230/115 C. 215/120 D. 185/110

21. During the administration of thrombolysis, blood pressure must be measured every 30 minutes

A. RIGHT B. WRONG C. DON'T KNOW

22. Administration of aspirin is recommended within 24 to 48 hours after thrombolysis

A. RIGHT B. WRONG C. DON'T KNOW

23 Thrombolysis can be administered to a stroke patient who is receiving a therapeutic dose of heparin

A. RIGHT B. WRONG C. DON'T KNOW

24. Maximum allowed dose of r-tPA is 40 mg<br>A. RIGHT B. WRONG C. DON'T KNOW
25. For thrombolysis to be administered, blood tests, chest x-ray and electrocardiogram must all be completed<br>A. RIGHT B. WRONG C. DON'T KNOW
26. After thrombolysis treatment, the patient must be transferred to an ICU for 12 hours<br>A. RIGHT B. WRONG C. DON'T KNOW
27. In a patient who has received thrombolysis, vital signs should be taken regularly only during the first 12 hours receiving r-tPA<br>A. RIGHT B. WRONG C. DON'T KNOW
28. In a patient with a severe stroke and large-vessel occlusion, thus with an indication for thrombectomy, we immediately administer thrombolysis (if the patient is eligible for that) and thrombectomy follows<br>A. RIGHT B. WRONG C. DON'T KNOW

Part C

**Self-assessment on knowledge of stroke recognition and management**				
How would you describe your level of knowledge on stroke in general?	Poorly	Well	Very well	Expert

ED: Emergency Department; EMS: Emergency Medical Services; ICU: Intensive Care Unit; NIHSS: National Institutes of Health Stroke Scale; MRI: Magnetic Resonance Imaging; r-tPA: recombinant-tissue Plasminogen Activator.

Supplementary File S2 (SF2): Multiple linear regression model predicting stroke knowledge as measured with the 28-item stroke knowledge questionnaire (N=281)

	**Total stroke knowledge score**				**General knowledge**				**Stroke recognition**				**Stroke management**			
**Independent variables**	**Beta**	**Std. error**	**t**	**p.value**	**B**	**Std. error**	**t**	**p.value**	**B**	**Std. error**	**t**	**p.value**	**B**	**Std. error**	**t**	**p.value**
**Hospital**																
Private Hospital A	-0.86	1.41	-0.61	0.544	-0.21	0.31	-0.68	0.499	-1.53	0.56	-2.74	**0.007**	-0.21	0.31	-0.68	0.499
Private Hospital B	-4.24	1.28	-3.31	**0.001**	-0.38	0.28	-1.37	0.172	-1.59	0.51	-3.16	**0.002**	-0.38	0.28	-1.37	**0.172**
Private Hospital C	-1.81	1.45	-1.24	0.215	-0.58	0.31	-1.84	0.067	-0.58	0.57	-1.01	0.315	-0.58	0.31	-1.84	0.067
Private Hospital D	-0.46	1.56	-0.29	0.768	-0.02	0.34	-0.05	0.960	-0.76	0.62	-1.22	0.223	-0.02	0.34	-0.05	0.960
Hospital A	-3.28	0.75	-4.39	**<0.001**	-1.13	0.16	-7.00	**<0.001**	-0.91	0.29	-3.10	**0.002**	-1.13	0.16	-7.00	**<0.001**
Hospital B	-2.58	0.95	-2.72	**0.007**	-0.40	0.21	-1.93	0.055	-0.64	0.38	-1.71	0.089	-0.40	0.21	-1.93	**0.055**
Hospital C	-1.37	1.05	-1.30	0.195	-0.31	0.23	-1.36	0.176	-0.93	0.42	-2.24	**0.026**	-0.31	0.23	-1.36	0.176
Hospital D	-3.85	0.95	-4.05	**<0.001**	-1.06	0.21	-5.17	**<0.001**	-0.86	0.38	-2.30	**0.022**	-1.06	0.21	-5.17	**<0.001**
Hospital E	-3.26	1.13	-2.89	**0.004**	-0.41	0.24	-1.70	0.091	-0.29	0.45	-0.66	0.511	-0.41	0.24	-1.70	0.091
Hospital F	-3.87	1.24	-3.11	**0.002**	-0.37	0.27	-1.35	0.177	-1.40	0.49	-2.84	**0.005**	-0.37	0.27	-1.35	**0.177**
**Gender**																
Female	0.04	0.50	0.09	0.930	0.08	0.11	0.76	0.449	0.12	0.20	0.59	0.557	0.08	0.11	0.76	0.449
**Age group (years)**																
30-39	-0.61	0.77	-0.80	0.423	0.00	0.17	0.00	1.000	0.05	0.30	0.18	0.856	0.00	0.17	0.00	1.000
40-49	-0.10	1.16	-0.09	0.930	0.48	0.25	1.91	0.057	0.12	0.46	0.27	0.790	0.48	0.25	1.91	0.057

50-60	-2.30	1.63	-1.41	0.160	-0.12	0.35	-0.35	0.726	-0.64	0.64	-0.99	0.324	-0.12	0.35	-0.35	0.726
>60	-1.23	3.12	-0.39	0.694	-0.42	0.68	-0.62	0.536	0.07	1.23	0.06	0.955	-0.42	0.68	-0.62	0.536
**Educational attainment**																
Diploma of Nursing School	0.10	2.17	0.05	0.964	0.21	0.47	0.45	0.650	-0.71	0.86	-0.82	0.411	0.21	0.47	0.45	0.650
Nursing specialty program	-1.37	2.36	-0.58	0.562	0.62	0.51	1.21	0.228	-1.30	0.93	-1.40	0.163	0.62	0.51	1.21	0.228
Bachelor Degree	-0.89	0.53	-1.66	0.098	-0.10	0.12	-0.84	0.400	-0.15	0.21	-0.72	0.473	-0.10	0.12	-0.84	0.400
**Profession**																
Physician	3.72	0.93	4.00	**<0.001**	0.22	0.20	1.11	0.269	1.28	0.37	3.48	**0.001**	0.22	0.20	1.11	0.269
**Work experience (in years)**																
1-5	3.99	1.77	2.26	**0.025**	0.70	0.38	1.82	0.069	1.07	0.70	1.53	0.127	0.70	0.38	1.82	0.069
6-10	3.78	1.82	2.07	**0.039**	0.54	0.39	1.37	0.171	0.77	0.72	1.08	0.283	0.54	0.39	1.37	0.171
11-20	4.47	1.91	2.33	**0.020**	0.48	0.41	1.17	0.245	1.01	0.76	1.33	0.185	0.48	0.41	1.17	0.245
21-30	5.15	2.27	2.27	**0.024**	0.36	0.49	0.73	0.464	1.33	0.90	1.48	0.139	0.36	0.49	0.73	0.464
>30	6.96	2.57	2.71	**0.007**	0.97	0.56	1.74	0.084	1.90	1.02	1.87	0.062	0.97	0.56	1.74	0.084
**Prior education/training in stroke care**	1.01	0.51	1.99	**0.048**	0.22	0.11	2.03	**0.044**	0.51	0.20	2.56	**0.011**	0.22	0.11	2.03	**0.044**
